# Acupuncture, Counseling, and Usual care for Depression (ACUDep): study protocol for a randomized controlled trial

**DOI:** 10.1186/1745-6215-13-209

**Published:** 2012-11-14

**Authors:** Hugh MacPherson, Stewart Richmond, J Martin Bland, Harriet Lansdown, Ann Hopton, Arthur Kang’ombe, Stephen Morley, Sara Perren, Eldon Spackman, Karen Spilsbury, David Torgerson, Ian Watt

**Affiliations:** 1 Department of Health Sciences, University of York, York YO10 5DD, United Kingdom; 2 University of Leeds, Leeds, LS2 9LJ, UK; 3 Centre for Health Economics, University of York, York, YO10 5DD, United Kingdom

**Keywords:** Depression, Acupuncture, Counseling, Primary care, Randomized controlled trial, Effectiveness, Cost-effectiveness

## Abstract

**Background:**

The evidence on the effect of acupuncture or counseling for depression is not conclusive yet is sufficient to warrant further research. Our aim is to conduct a full-scale RCT to determine the clinical and cost effectiveness of acupuncture and counseling compared to usual care alone. We will explore the experiences and perspectives of patients and practitioners.

**Methods/Design:**

Randomized controlled trial with three parallel arms: acupuncture plus usual care, counseling plus usual care, and usual care alone, in conjunction with a nested qualitative study using in-depth interviews with purposive samples of trial participants.

Participants: Patients aged over 18 years diagnosed with depression or mood disorder by their GP and with a score of 20 or above on the Beck Depression Inventory (BDI-II).

Randomization: Computer randomization by York Trials Unit to acupuncture, counseling, and usual care alone in proportions of 2:2:1, respectively, with secure allocation concealment.

Interventions: Patients allocated to acupuncture and counseling groups receive the offer of up to 12 weekly sessions. Both interventions allow flexibility to address patient variation, yet are constrained within defined protocols. Acupuncture is based on traditional Chinese medicine and counseling is non-directive within the humanistic tradition.

Outcome: The PHQ-9 is the primary outcome measure, collected at baseline, 3, 6, 9, and 12 months. Also measured is BDI-II, SF-36 Bodily pain subscale, and EQ-5D. Texted mood scores are collected weekly over the first 15 weeks. Health-related resource use is collected over 12 months.

Analysis: The sample size target was for 640 participants, calculated for an effect size of 0.32 on the PHQ-9 when comparing acupuncture with counseling given 90% power, 5% significance, and 20% loss to follow-up. Analysis of covariance will be used on an intention-to-treat basis. Thematic analysis will be used for qualitative data. We will compare incremental cost-effectiveness of the three treatment options at 12 months.

**Discussion:**

Ethical approval was obtained in October 2009. There were six subsequent protocol amendments, the last of which was approved in January 2012. Recruitment of 755 participants took place over 18 months. Data collection will be completed by June 2012. No interim analyses have been conducted.

**Trial registration:**

ISRCTN63787732

## Background

Depression will be the second leading cause of disease burden worldwide by 2020 according to the Global Burden of Disease Study [1]. As of 2000, an estimated 2.6 million cases of depression were reported in England [2], and it is the third most common reason for primary care consultations [3]. The economic burden in England is estimated to exceed £9 billion per annum with approximately £370 million going to direct costs of treatment [2]. As many as 50% of people with depression also have chronic pain [4]. Despite these considerable costs, current pharmacological and psychological interventions have limited acceptability and effectiveness. Up to 60% of patients do not adequately respond to pharmacological antidepressant treatment [5] and 30% do not adhere to medication [3]. Patients have expressed the view that there is an over-reliance on prescribed antidepressant medications [6] and they are keen to have a range of possible treatment choices [7]. More recently doubts have been cast on the effectiveness of newer anti-depressants, particularly in their effectiveness in lower severity depression [8].

Depression is commonly treated by acupuncturists [9] although this is rarely provided within NHS mental health services or primary care [10]. The evidence base from systematic reviews suggests that there could potentially be benefits from acupuncture, with sufficient evidence to warrant further research [11-13]. Counseling is a credible and widely used intervention for patients with depression, with approximately half of the 9,000 GP practices in England employing a counselor [14].

We have conducted two exploratory studies that are relevant to this proposal. The first study used mixed methods to explore the potential role of acupuncture for depression, using a focus group and a small uncontrolled outcome study [15]. The focus group of patients who had experienced depression helped shape our research. The second study was funded as part of a post-doctoral fellowship by the NIHR National Co-ordinating Centre for Research Capacity Development. We first conducted 30 in-depth interviews with patients, acupuncturists, and general practitioners (GPs). These qualitative data informed our design of a pilot RCT, including our choice of patient group, of outcome measures and of comparator interventions. We then conducted a pilot randomized controlled trial where we compared acupuncture plus usual GP care with counseling plus usual care with usual GP care alone for patients with depression (ISRCTN 59267538) [16]. Forty patients were recruited from two GP practices in York. The main outcome measures used were the Beck Depression Inventory (BDI-II) [17] and the Patient Health Questionnaire-9 (PHQ-9) [18]. When comparing BDI outcomes between acupuncture and counseling and usual care alone at 3, 6, and 9 months, we found a growing trend in favor of acupuncture and counseling. Equivalent changes were observed on the PHQ-9. More importantly, we established the feasibility of recruitment and identified key features including the sample size required for a full-scale trial.

For the proposed trial, the primary aim is to evaluate the clinical and cost effectiveness of acupuncture and counseling when offered in primary care as an adjunct to usual GP care. Our secondary aim is to compare acupuncture to counseling. Our design with both acupuncture and counseling arms in the one trial is supported by the recent Cochrane systematic review of acupuncture for depression that suggested: ‘Future studies may need to consider the use of comparative designs using medication or structured psychotherapies (cognitive behavioral therapy, psychotherapy, counseling) or standard care, due to the ethics of administering this intervention to this study population’ [11]. We will also be conducting health economic evaluations to determine the cost-effectiveness of acupuncture and counseling.

### Aims

The primary aim is to determine the clinical and cost effectiveness of short courses of acupuncture or counseling for depression when compared to usual GP care. Important secondary aims are: (a) to determine whether acupuncture is more effective than counseling or *vice versa*; and (b) to compare the experiences of patients, therapists, and GPs who took part in the trial in order to identify any relevant issues concerning acceptability, appropriateness, and feasibility in relation to future treatment provision and research.

## Methods

### Design

A randomized controlled trial will be conducted for patients with depression who will be allocated to one of three arms: acupuncture plus usual care, counseling plus usual care, and usual care alone, in conjunction with a nested qualitative study with purposive samples of patients and practitioners participating in the trial.

### Inclusion/exclusion criteria

We will recruit patients aged over 18 years who have been diagnosed with depression or mood disorder and: (a) have consulted their GP for depression within the past 5 years; or (b) are currently in receipt of a prescription for anti-depressant medication. Participants must also still be depressed at the time of recruitment, with a score of 20 or above on the Beck Depression Inventory-II (the conventional cut-offs used are: 0 to 13 for minimal depression; 14 to 19 for mild depression; 20 to 28 for moderate depression; and 29 to 63 for severe depression) [18]. We will exclude patients who are receiving acupuncture or counseling sessions, who have terminal illness, hemophilia, hepatitis, HIV, are pregnant, or who have a confounding psychiatric condition (bipolar disorder, postpartum depression, adjustment disorder, psychosis, or personality disorder). Patients who have suffered a close personal bereavement or have given birth during the previous 12 months will not be eligible to take part. Because of the specific demands of this study in terms of the need to fully comprehend detailed information, to complete questionnaires, to understand and converse readily with English speaking acupuncturists and counselors, and to permit physical contact, we intend to exclude patients who have a significant learning disability, who are unable to converse in English, or who have dementia.

### Randomization

Consenting patients will be allocated remotely by the York Trials Unit, with the allocation code concealed from the recruiting researcher, to the three groups in the proportions of 2:2:1 to acupuncture, counseling, and usual care alone, respectively.

### Outcome measures

The primary clinical outcome is the PHQ-9 [19] at 3 months, as an impact at this time-point is important to patients, and the primary economic outcome is at 12 months. An evaluation of whether there is an overall clinical benefit to patients over 12 months is also of considerable importance, and this will be evaluated in terms of PHQ-9 scores in the first secondary analysis of clinical outcomes. We will use the correlation between the PHQ-9 and BDI-II at baseline and 12 months to determine equivalent BDI-II scores at subsequent time points, in the context that a three-point difference between groups on the BDI-II is considered clinically significant [6]. As an additional measure we will use the SF-36 Bodily pain subscale [20]. All patients who agree to be contacted by text messaging will be asked for a single mood score (from 1 to 9) once a week for the first 15 weeks, in order to plot the trajectory of change over time, and they will be reimbursed. As a utility measure, the EQ-5D will be also be used [21]. Data will be collected at baseline, 3, 6, 9, and 12 months. At baseline we will collect demographic data as well as prior preferences and expectations of patients. For both acupuncture and counseling groups, we will measure the patient’s perception of the practitioners’ empathy using the Consultational and Relational Empathy (CARE) measure at 3 months, which we have tested with acupuncture patients [22]. Therapists will record in logbooks the number and length of times of sessions, the treatment provided, and any adverse events. At 3, 6, 9, and 12 months we will collect data on the impact of depression on work and normal activities. We will collect data on NHS resource use and patients’ private costs, to include additional contacts with acupuncturists and counselors, and over-the-counter medication. At 3 months, we will randomize the overall group into two and send to one group a text message timed to arrive the day before the questionnaire, reminding the recipients that the questionnaire will arrive and asking that they return them promptly. At 6 months the participants will be randomized into two groups, one will receive the text message before receiving the questionnaire and the other within the week following receipt of the questionnaire. At 9 months we will randomize the group into two and one group will be sent a text message reminder within the week following the arrival of the questionnaire. If a text message either before or after proves to enhance the response rate or time to response, we will send reminder texts to all patients to maximize response at the 12-month follow-up. A £5 note or voucher will be enclosed with the final questionnaire, as reimbursement is known to enhance response rates [23].

Using in-depth interviews, qualitative data will be collected from a purposive sample of patients to gain understanding of their perceptions and experiences of acupuncture, counseling, and usual GP care using interview schedules with topic guides. We also explore patients’ preferences, beliefs, and understanding of their depression, and the impact of these factors might have on their experience of treatment and subsequent outcome.

After patient recruitment has been completed, we will explore the experiences of acupuncturists, counselors, and GPs who took part in the trial. This will involve conducting a series of in-depth interviews and focus groups with individuals drawn from these three professional groups. These nested qualitative studies will aim to provide insights that may enhance standards of practice in relation to the treatment of depression, guide the development of future randomized controlled trials involving patients with depression, and identify implications for health policy.

### Interventions

Patients allocated to the acupuncture and counseling groups will receive the offer of 12 sessions on a weekly basis. The participating acupuncturists will be members of the British Acupuncture Council who have been qualified for at least 3 years. The acupuncture treatments will be performed according to a treatment protocol already developed for this purpose [24] and tested in our pilot. This will allow for each patient having a customized treatment within a standardized theory-driven framework. Counseling will be provided by members of the British Association of Counseling and Psychotherapy using primarily a non-directive approach [25]. A manualized protocol, which was developed for the pilot, will set the parameters for the counseling. Counselors will use empathy and advanced listening skills to help clients express feelings, clarify thoughts, and reframe difficulties, but they will not give advice or set homework. Variations in treatment of patients by counselors and acupuncturists will be reported in order to better interpret findings and assess generalizability. Usual GP care will continue to be available to all patients according to need, and will be monitored and reported across all three groups for comparative purposes, and this to include acupuncture and counseling received, whether allocated within the trial or not.

### Sample size

We will test for an effect size of 0.32 on the PHQ-9 when comparing acupuncture and counseling (1:1) with 90% power and 5% significance, which will require 204 per group. We will test for an effect size of 0.39 when comparing either acupuncture or counseling to usual care alone (2:1), with the usual care group being half the size of the acupuncture and counseling groups, for which we will need 103 patients in the usual care group. Allowing for 20% attrition, and randomizing in the proportions 2:2:1 for acupuncture, counseling, and usual care, respectively, the total sample size required is 640 (that is, groups of 256, 256, and 128, respectively).

One hundred purposively selected patients will be invited to participate in-depth interviews using a topic guide, the number to be decided when saturation has been reached. Forty percent of them (that is, 40 participants) will be drawn from the group allocated to acupuncture, another 40% from those allocated to counseling, and 20% (that is, 20 participants) will be from those allocated to usual care alone. In order to capture and map the diversity, a sampling frame will be used with sampling criteria based on sex and baseline scores. In addition, up to 65 therapists/GPs will be recruited to take part in a qualitative sub-study, which will explore their experiences of delivering the trial intervention to patients with depression.

### Recruitment

Using GP databases for identification and screening, and based on our finding from our pilot where we recruited 40 patients from GP databases with a combined list of 21,500, we estimate that we will recruit 0.19% of patients from GP lists. To recruit 640 patients, we estimate a total primary care list size of approximately 337,000 patients is required, equivalent to 22 GP practices with an average list of 15,000 patients each. We have planned an 18-month recruitment period. We will work closely with the Primary Care Networks to recruit GP practices, ensuring they are located nearby clusters of suitable therpaists.

Acupuncturists, counselors, and GPs who agree to support the trial will be invited to take part in a qualitative sub-study after patient recruitment has ended. Each will receive a participant information sheet and consent forms, which will be different from those sent to patients.

### Analysis

We will compare three clinical outcomes: (1) acupuncture plus usual care *versus* usual care alone; (2) counseling plus usual care *versus* usual care alone; and (3) acupuncture plus usual care *versus* counseling plus usual care.

A pre-specified statistics analysis plan will be used. Analysis will be on an intention-to-treat basis. We will use analysis of covariance at 3 months and the ‘area under the curve’ method at 12 months to assess whether clinical differences, if any, are significant. In a secondary analysis, when comparing acupuncture with counseling ((3) above), and to better understand the treatment effect, we will control for treatment time (number and length of sessions) and empathy (patient’s score of their practitioner’s empathy) by including them as covariates in the analysis. Methods to deal with potentially missing data will be addressed in detail in the analysis plan. The proportion of missing data will be summarized at each time point by allocated treatment, and known reasons for missing data will be summarized. Multiple imputation based on the standard best-subset regression method will be used for missing data. We will also estimate whether prior expectations or preferences are effect modifiers [26].

We will explore practitioner effects by putting a group of acupuncture patients and a matching number of controls in the same cluster and use a single summary statistic, difference between acupuncture and control means, for that cluster. We will then estimate the mean difference across clusters using the t method for the confidence interval and to test the null hypothesis that the mean difference is zero. This will deal with the problem that between cluster variability is expected to be present for acupuncture patients but not for controls. It will provide an estimate of the variation between practitioners in treatment effect as well as an estimate of the treatment effect which allows for practitioner variation.

For the qualitative data, we will audiotape the interviews (with the permission of participants) and subsequently transcribe verbatim. Qualitative data will be coded and analyzed thematically within the framework of illness narratives [27,28] and thematic analysis [29]. This will assist in the interpretation of the quantitative data from the trial, and provide a better understanding about who is likely to be attracted to and/or benefit from prescribed medication, from acupuncture, and from counseling.

An initial analysis of transcribed data from interviews with therapists and GPs will be conducted jointly by two members of the research team to identify emerging themes and issues, in particular those which would merit further exploration in the focus groups. These emerging findings will be reviewed and developed by the full research team (where appropriate drawing on learning from the main trial) and will be used to shape the topic guides for the focus groups. Once the focus group phase has been completed, the transcripts from all the interviews and focus groups will be analyzed thematically. The main areas of inquiry covered in the interview schedules/topic guides will initially be used as a framework for analyzing the data across all three groups of professionals contributing to the study. However, within each main area an ‘inductive’ process will be used to identify emergent themes and issues [29], and to explore shared experiences or points of difference between the three professional groups. As this study is intended to inform future treatment of patients with depression, the analysis will also seek to draw out findings which might inform the practice of acupuncturists and counselors, and wider service delivery for patients with depression. During the analysis, regular meetings will be held by the research team to check and shape the themes and to ensure that any insights gained from the main trial data are incorporated into this sub-study. Findings derived from interviews and focus groups with participating patients, acupuncturists, counselors, and GPs will then be used collectively to develop recommendations for future research, policy, and practice in relation to the treatment of depression.

The economic analysis will consist of a within trial analysis and an extrapolation of trial outcomes to longer-term time horizons incorporating other relevant evidence. For the within trial analysis we will compare the three treatment options based on the NHS costs and effectiveness as measured by the EQ-5D [21] over the trial follow-up period. Decision analytic modeling will be used to extend the trial analysis to the life-time of the patient and include other relevant information. The incremental cost-effectiveness ratio will be calculated from the estimation of long-term costs and QALYs. Probabilistic sensitivity analysis will be conducted to allow for a full representation of parameter uncertainty [30].

## Discussion

**Figure 1:**
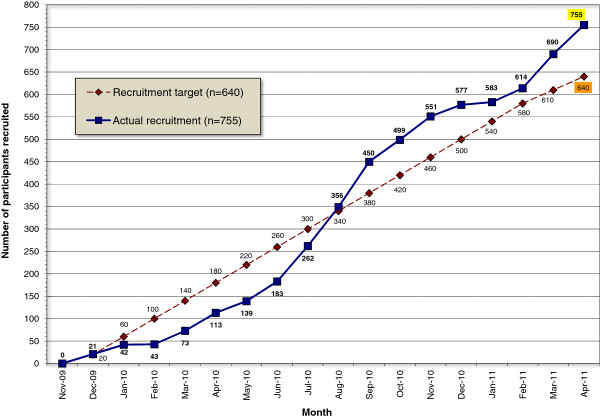
**Recruitment of participants into the ACUDep trial (Acupuncture, Counseling and Usual GP Care for Depression).** Total number of participants recruited = 755.

An initial version of this protocol was approved by York NHS Research Ethics Committee (Ref no: 09/H1311/75) on 1 October 2009. A series of six protocol amendments were subsequently approved, the final one on 4 January 2012. In total, 755 patients have been recruited to the trial over an 18-month period between November 2009 and April 2011, see Figure 1. The last of the 100 patients will be recruited to the qualitative sub-study by June 2012. Data collection will be completed by the end of June 2012. Data entry will be completed by the end of August. No interim analyses have been conducted. Analysis of clinical and health economic data will start in September and is expected to be complete by the end of 2012, followed by submission of primary results to a peer-reviewed journal in 2013.

## Trial status

Closed as of June 2012.

## Competing interests

The authors declare that they have no competing interests.

## Authors’ contributions

HM, JMB, SM, DT, and IW helped design the trial as part of their role as co-applicants on the Programme Grant for Applied Research which is funding this trial. SR as Trial Manager has had input into features of the trial design and co-ordinated the management of the trial. AK and ES contributed to the statistical and health economic analysis plans, respectively. HL and SP contributed to the design and development of the treatment protocols for the acupuncture and counseling, respectively. AH and KS contributed to the design and conduct of the qualitative components of the study. All authors read and approved the final manuscript.
